# The Best Screening Test Is the One That Gets Followed-Up On

**DOI:** 10.1053/j.gastro.2026.02.013

**Published:** 2026-02-23

**Authors:** DANICA M. N. VAN DEN BERG, CHIARA C. BRÜCK, PEDRO NASCIMENTO DE LIMA, FERNANDO ALARID-ESCUDERO, ANNE I. HAHN, IRIS LANSDORP-VOGELAAR, COLORECTAL WORKING GROUP OF THE CANCER INTERVENTION AND SURVEILLANCE MODELING NETWORK (CISNET)

**Affiliations:** Department of Public Health, Erasmus MC University Medical Center, Rotterdam, the Netherlands; Department of Public Health, Erasmus MC University Medical Center, Rotterdam, the Netherlands; RAND Corporation, Arlington, Virginia; Department of Health Policy, School of Medicine, Stanford University, Stanford, California, and Stanford Health Policy, Freeman-Spogli Institute for International Studies, Stanford University, Stanford, California; Department of Epidemiology and Biostatistics, Memorial Sloan Kettering Cancer Center, New York, New York; Department of Public Health, Erasmus MC University Medical Center, Rotterdam, the Netherlands

The well-known saying by Dr Sidney Winawer, “the best test is the test that gets done and done well,”^1^ highlights that screening is only effective when the screening test is both completed and performed correctly, emphasizing the crucial role of the acceptability and quality of screening procedures. Guided by this principle, numerous initiatives have focused on increasing colorectal cancer (CRC) screening uptake and improving test quality. One notable example is the “80% by 2018” campaign in the United States, launched by the National Colorectal Cancer Roundtable and the American Cancer Society in 2014, which aimed to increase CRC screening rates to 80% among adults 50 years of age and older by 2018.^2^ Although the goal of 80% was not reached, screening participation rates have increased from 65% before the campaign (2012) to almost 70% after the campaign (2020).^2^ Recently, blood-based screening tests have emerged as promising triage tools to enhance screening uptake, particularly among populations that would not otherwise use existing screening methods. However, these efforts overlook the fact that there is no benefit of screening without follow-up. Without timely and appropriate follow-up, the potential benefits of screening are negated, and individuals may only incur the harm associated with the incomplete screening process.

## The CRC Screening Pathway: A Leaky Pipeline?

**Figure 1.**
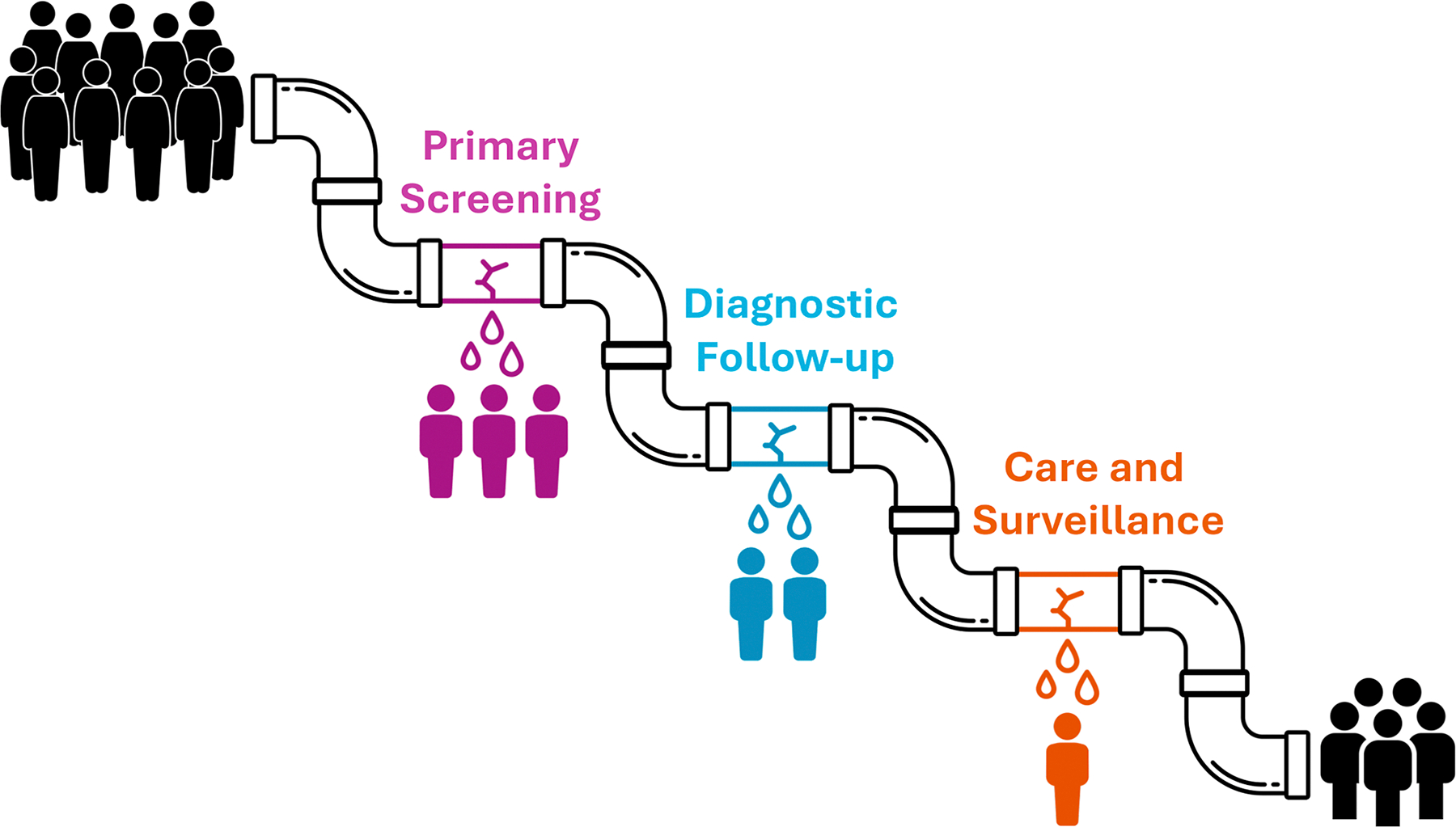
The leaking colorectal cancer screening pipeline.

CRC screening is recommended by guidelines in many countries, such as those from the United States Preventive Services Task Force, which advises average-risk adults 45 to 75 years of age to undergo regular screening.^3^ CRC screening can be performed in two main ways: (1) through a colonoscopy, which serves as both a screening and diagnostic procedure; or (2) using non–colonoscopy-based tests (eg, stool-based and blood-based tests), which require a diagnostic follow-up colonoscopy if results are abnormal. Unfortunately, although much attention is placed on adherence to primary screening, many individuals are lost to follow-up after receiving an abnormal stool or blood test result, causing a leaky pipeline in the CRC screening pathway (Figure 1). The Multi-Society Task Force recommends that at least 80% of positive fecal immunochemical tests (FITs) be followed up with colonoscopy.^4^ However, adherence to this benchmark varies widely across and within countries. In Europe, follow-up rates after a positive FIT tend to be high in many organized population-based screening programs with centralized invitation, tracking, and recall systems, with an average completion of 81% (range, 64%–92%). In contrast, non–population-based programs report substantially lower follow-up completion, at approximately 50%.^5^ In the United States, the situation is more concerning: almost half of the individuals (44%) do not complete a colonoscopy within 1 year after a positive stool-based test,^6^ with substantial variability across health care settings. Integrated health systems such as Kaiser Permanente achieve follow-up rates exceeding 85%,^7^ comparable to those observed in organized European programs. In contrast, safety-net health systems, which provide care primarily for low-income, vulnerable, or underinsured and uninsured populations and rely heavily on public funding, report follow-up rates below 45%.^8^

## Importance of Follow-Up

Evidence from multiple modeling studies indicates that without a prompt diagnostic colonoscopy, the benefits of early detection are significantly reduced. Meester et al^9^ estimated that a delay of up to 12 months following a positive FIT could result in a nearly 10% loss of the overall benefit of screening. Similarly, Rutter et al^10^ showed that longer times to diagnostic testing after an abnormal screening result significantly decrease the effectiveness of screening. More recently, Fendrick et al^11^ expanded this modeling work by examining how adherence to follow-up affects the reduction in CRC incidence and mortality associated with annual FIT screening and triennial multitarget stool DNA (mt-sDNA) screening. Their findings indicate that, under imperfect follow-up adherence (47% for FIT and 73% for mt-sDNA), the reduction in CRC incidence falls from 50% to 23% for FIT and from 61% to 43% for mt-sDNA, compared with scenarios assuming complete follow-up. Similarly, the reduction in CRC mortality declines from 59% to 27% for FIT and from 69% to 49% for mt-sDNA when imperfect follow-up rates are assumed instead of 100% follow-up. A separate analysis simulating adherence and follow-up rates observed in safety-net systems showed that a screening program using blood-based tests would not be cost-effective with imperfect follow-up rates even compared to no screening, demonstrating how important adherence is for the cost-effectiveness of screening modalities.^12^

We further show the importance of follow-up adherence with a small modeling exercise, using the microsimulation model MISCAN-Colon. This model simulates the life histories of a large population of individuals from birth until death. As each simulated person ages, adenomas may develop and grow over time, potentially progressing to CRC. In addition to the natural history of CRC, MISCAN-Colon can simulate different screening programs for the early detection of CRC. The modeling set-up of this exercise has previously been described.^13^

In this exercise, we simulated a population of 10 million individuals, each of whom was offered a one-time FIT screening at 45 years of age. Supplementary Table 1 provides an overview of the test characteristics used in the analysis, consistent with those in earlier modeling studies.^13^ We varied adherence rates for both the initial FIT screening and the follow-up colonoscopy across five levels: 0%, 25%, 50%, 75%, and 100%. The resulting scenarios were evaluated based on the number of life years gained compared to the baseline scenario, which simulated no screening.

**Figure 2.**
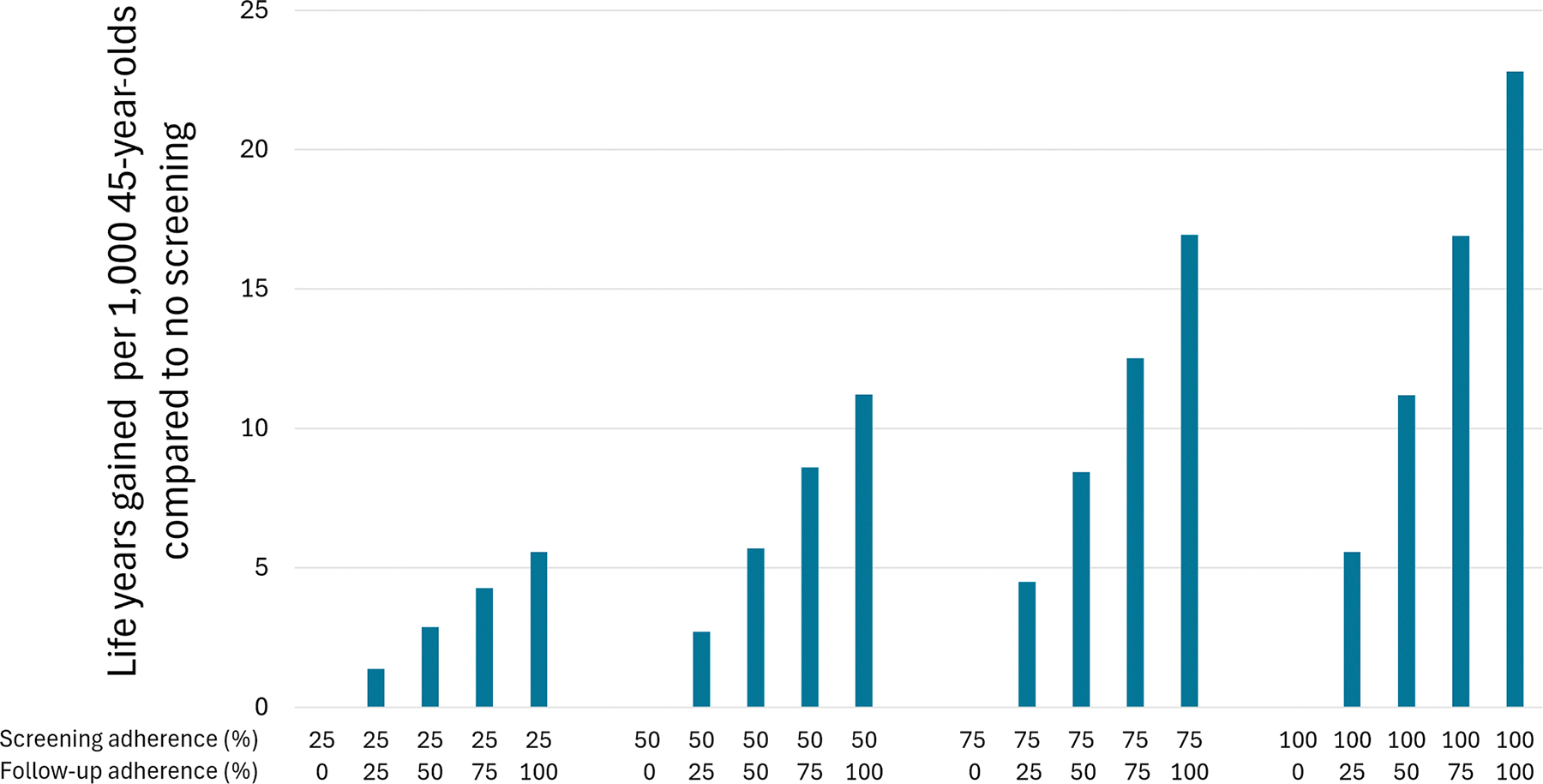
Life years gained per 1000 individuals screened at age 45 with fecal immunochemical test compared to no screening.

The results of the simulation exercise explicitly demonstrate that the effectiveness of screening depends as much on adherence to diagnostic follow-up colonoscopy as on participation in primary screening. As shown in Figure 2, scenarios with 0% follow-up adherence yield no additional life years compared to no screening, regardless of the initial screening uptake. This indicates that without follow-up confirmation, the potential benefits of early detection are entirely lost. Increasing follow-up adherence leads to substantial gains in life years, even when screening adherence remains constant, and these gains tend to increase linearly with follow-up adherence at each level of screening uptake. Interestingly, the results suggest that higher follow-up adherence can compensate for lower screening adherence. For example, with the FIT and colonoscopy characteristics currently observed in the United States, the scenario with 25% screening adherence and 75% follow-up adherence yields a similar number of life years gained as the reverse scenario with 75% screening adherence and 25% follow-up adherence. This highlights that follow-up adherence is equally, if not more, important than initial screening adherence to the overall effectiveness of the program, because high screening uptake without adequate follow-up still exposes individuals to the costs and potential harms of screening without delivering any health benefit.

Beyond modeling studies, clinical data confirms these findings. A cohort study of patients within the Kaiser Permanente health care system in the United States found that individuals who did not follow up on abnormal FIT results had a seven-fold higher risk of CRC death than those with a timely follow-up colonoscopy.^14^ Similarly, a European study reported that non-compliance with colonoscopy after a positive FIT doubles the risk of dying from CRC.^15^ Another study examined how the interval between a positive FIT and subsequent colonoscopy affected CRC risk in patients enrolled in Kaiser Permanente’s organized screening programs.^7^ They found that delaying follow-up colonoscopy beyond 9 months was associated with a significantly increased risk of CRC (odds ratio, 1.48; 95% CI, 1.05–2.08).

## Barriers to Successful Follow-Up

Despite clear recommendations for follow-up after abnormal stool- or blood-based screening tests, multiple barriers hinder individuals from completing this critical step. These barriers exist at both the health system and individual levels.

At the health system level, limitations in care coordination and infrastructure significantly hinder follow-up. Poor communication between screening sites and diagnostic services, combined with the absence of tracking systems or reminder protocols, contribute to patients being lost to follow-up.^16^ In addition, inappropriate screening may contribute to low observed follow-up rates. Screening is sometimes performed in individuals with significant comorbidities or limited life expectancy for whom colonoscopy may be unsafe or no longer appropriate, resulting in a positive screening test without a feasible diagnostic pathway.^17^ Access to specialists is another critical bottleneck. Long wait times and a shortage of gastroenterologists, projected to reach a deficit of more than 1500 providers in 2025 in the United States,^18^ limit the availability of timely colonoscopy appointments. Reimbursement and insurance-related challenges further complicate the follow-up process. Although recent policies have eliminated cost-sharing for follow-up colonoscopies in some cases,^19^ many patients still face high out-of-pocket expenses, particularly if they are uninsured or underinsured. In one study, 38% of patients who did not complete follow-up colonoscopy cited insurance-related challenges as the primary reason.^20^

Patients also face a range of individual challenges. A common barrier is a limited understanding of the screening process. Many patients are unaware of the implications of a positive stool- or blood-based test and do not realize that a follow-up colonoscopy is essential to complete the screening cycle. Azulay et al^21^ found patient lack of comprehension regarding the test was the strongest predictor of non-adherence to follow-up (odds ratio, 0.52; 95% CI, 0.37–0.71). Low health literacy and language barriers further hinder patient follow-up rates.^16^ In addition to informational barriers, psychological and emotional factors play a significant role. Fear of a cancer diagnosis, anxiety about the colonoscopy procedure, and concerns about discomfort or complications were frequently cited as barriers, particularly among individuals in safety-net settings.^8^ Cultural perceptions also play a role; for instance, colonoscopy has been described as stigmatizing or emasculating by some male patients, leading to avoidance.^22^ Logistical challenges are another major obstacle. Many patients face difficulties arranging transportation, securing child care, or taking time off work as well as the requirement to have a companion after sedation reduces adherence to follow-up colonoscopies.^16^ A recent microsimulation study found that implementing a rideshare program to address transportation barriers was cost-saving up to $100 per ride by improving follow-up after abnormal FIT results, leading to earlier detection and cancer prevention.^23^

## Conclusions and Future Directions

Follow-up colonoscopies must be considered an integral part of the screening process to fully achieve the life-saving potential of CRC screening. A positive stool- or blood-based test does not mark the conclusion of screening; rather, it indicates the need for a timely follow-up colonoscopy to attain meaningful clinical benefit for the patient. Public messaging should shift from promoting primary CRC screening tests in isolation, and rather emphasize the entire screening pipeline, making clear that screening is complete only when appropriate follow-up is provided. To ensure patients are not lost along the screening pipeline, system-level interventions such as patient navigation, reminder systems, and care coordination infrastructure are essential.

The importance of adherence to a follow-up confirmatory test is increasingly recognized in various quality assessments of cancer screening programs. For example, in a recent Delphi study, involving 33 cancer screening experts, follow-up adherence was acknowledged as essential to the quality of a screening program.^24^ Health care quality measures, such as the Healthcare Effectiveness Data Information Set (HEDIS) from the United States National Committee for Quality Assurance (NCQA), have previously focused primarily on adherence to initial screening. However, in 2025, NCQA recognized the importance of follow-up adherence and announced that a new HEDIS measure is in development, which will also focus on follow-up adherence.^25^ This expansion of quality measures to include follow-up adherence is a promising first step toward aligning incentives with outcomes.^25^

Although this commentary focuses on the importance of follow-up adherence in CRC screening, follow-up is a critical component of any disease screening process that involves a primary screening test followed by a confirmatory diagnostic test. For example, in cervical cancer screening, a Pap smear or another screening test for the human papillomavirus (HPV) serves as the primary screening method, and the samples of individuals who are HPV positive are tested for cellular changes. If abnormal cells are found, the individual is referred for colposcopy to evaluate further and confirm the findings. Therefore, adherence to follow-up after an abnormal screening result is essential to ensure that the benefits of early detection are fully realized across different disease screening programs.

Building on earlier insights that emphasize the importance of screening participation and test quality, we should now recognize that “the best test is the test that gets followed up on,” because without timely diagnostic evaluation, even perfect primary test adherence and high-quality screening fail to deliver the potential benefits of early detection.

## Supplementary Material

1

Note: To access the supplementary material accompanying this article, visit the online version of *Gastroenterology* at www.gastrojournal.org, and at https://doi.org/10.1053/j.gastro.2026.02.013.

## Data Availability

Model results and Python code for figures are available upon reasonable request to the corresponding author.
