# Supplementary material for: The Best Screening Test Is the One That Gets Followed-Up On
Source: Gastroenterology. Author manuscript; Available in PMC 2026 Aug 4. (PMC13435187; doi:10.1053/j.gastro.2026.02.013)
Supplement: 1 [file NIHMS2190402-supplement-1.pdf]

Supplemental material with the manuscript

The best screening test is the one that gets followed up on

Danica MN van den Berg, Chiara C Brück, Pedro Nascimento de Lima, Fernando Alarid-Escudero, Anne I Hahn, Iris Lansdorp-Vogelaar; Colorectal Working Group of the Cancer Intervention and Surveillance Modeling Network (CISNET)

Supplemental Table 1 provides an overview of the test characteristics used in the analysis, consistent with those in earlier modeling studies.[1]

Supplemental Table 1 Screening Test Characteristics Used in the Analysis

	Specificity	Sensitivity				Source
		Adenomas 1 to <6 mm	Adenomas 6 to <10 mm	Adenomas ≥10 mm	CRC	
**FIT**	0.964	0.076	0.076	0.238	0.738	[2]
**Colonoscopy**	1	0.69	0.81	0.91	0.91	[3, 4]

References:

1. van den Puttelaar R, Nascimento de Lima P, Knudsen AB, et al. Effectiveness and Cost-Effectiveness of Colorectal Cancer Screening With a Blood Test That Meets the Centers for Medicare & Medicaid Services Coverage Decision. Gastroenterology 2024;167:368-377.
2. Lin JS, Perdue LA, Henrikson NB, et al. Screening for colorectal cancer: updated evidence report and systematic review for the US Preventive Services Task Force. Jama 2021;325:1978-1998.
3. Schroy Iii PC, Coe A, Chen CA, et al. Prevalence of advanced colorectal neoplasia in white and black patients undergoing screening colonoscopy in a safety-net hospital. Annals of internal medicine 2013;159:13-20.
4. Zhao S, Wang S, Pan P, et al. Magnitude, risk factors, and factors associated with adenoma miss rate of tandem colonoscopy: a systematic review and meta-analysis. Gastroenterology 2019;156:1661-1674. e11.
